# GloveCare: a pilot study in preparation for a cluster crossover randomized controlled trial of non-sterile glove-based care in preventing late-onset infection in the NICU

**DOI:** 10.1186/s40814-023-01271-9

**Published:** 2023-03-23

**Authors:** Sarah Khan, Kara K. Tsang, Zheng Jing Hu, Beata Mostowiak, Salhab El Helou, Michelle Science, David Kaufman, Jeffrey Pernica, Lehana Thabane, Dominik Mertz, Mark Loeb

**Affiliations:** 1 McMaster University, Hamilton, Canada; 2 McMaster Children’s Hospital, 1200 Main St. West, 3A, Hamilton, ON Canada; 3 London School of Tropical Medicine and Hygiene, London, UK; 4 University Health Network, Toronto, Canada; 5 University of Toronto, Toronto, Canada; 6 University of Virginia, Charlottesville, VA USA

**Keywords:** Late-onset infection, Nonsterile gloves, Neonatal intensive care unit

## Abstract

**Background:**

Late-onset infections (LOI) are a major cause of morbidity and mortality among patients in the neonatal intensive care unit (NICU). Gloving after hand hygiene may be a pragmatic approach to prevent infections that arise when healthcare workers’ hands transmit pathogens to neonates.

**Objective:**

To determine the feasibility of conducting a multicenter, open-labeled randomized controlled trial (RCT) to determine whether a protocol that requires healthcare workers (HCWs) in a level 3 NICU to wear non-sterile gloves plus hand hygiene reduces the occurrence of a late-onset infection, compared to hand hygiene alone.

**Methods:**

In this single-center pilot study, we recruited neonates admitted to the McMaster Children’s Hospital NICU from June 2017 to May 2018. The NICU was randomized to begin with the standard (control) arm for 6 months (June 2017 to Dec 2017), followed by the gloving (GloveCare) arm for 6 months (Jan 2018 to July 2018), with a 2-week washout period in-between to educate healthcare workers about gloving. We measured numerous feasibility outcomes including enrollment, event rate, and compliance with hand hygiene (Moment 1: before patient contact, Moment 2: before clean procedure, Moment 3: after body fluid contact, Moment 4: after patient contact) and gloving compliance.

**Results:**

We enrolled 750 neonates (390 Standard care, 360 GloveCare) and achieved 100% enrollment. We found higher hand hygiene compliance during the standard care arm compared to the GloveCare for all four moments of hand hygiene (Moment 1: 87% vs 79%, OR=1.86 (1.34, 2.59); Moment 2: OR=1.73 (1.00, 3.01); Moment 3: OR=1.11 (0.62, 1.98); Moment 4: OR=1.65 (1.27, 2.14)). We developed and validated a method to calculate glove compliance, which ranged from 48 to 85%, and was highest for moment 3 (doffing after a procedure or body fluid exposure risk). No adverse events were documented for patients or staff.

**Discussion:**

Reduction in hand hygiene compliance in the GloveCare arm presents a pragmatic challenge in ascertaining the effectiveness of gloving to prevent LOI. Most LOIs were non-sterile-site infections, which is considered a less patient-important or clinically relevant outcome compared to sterile-site LOI. Ensuring efficient collection and validation of hand hygiene and gloving data is imperative.

**Conclusion:**

The pilot study demonstrated the feasibility of this intervention though modifications to improve hand hygiene compliance during GloveCare will be important prior to a multicenter cluster RCT to assess the efficacy of non-sterile glove-based care in preventing LOI in the NICU.

**Trial registration:**

Clinicaltrials.gov, NCT03078335

## Key messages regarding feasibility

- What uncertainties existed regarding the feasibility?
  The feasibility of implementing, sustaining, and measuring compliance with glove-based care has not been studied in a systematic way before. In order to roll this intervention out in the multicenter-based trial required to demonstrate a difference in late-onset infection, the feasibility of implementing this intervention is critical to the fidelity of a future pragmatic trial.
- What are the key feasibility findings?
  Our participant enrollment (target >90%) feasibility outcome was met through the use of waiver of consent and an opt-out of data collection option. We did not achieve our target of 90% hand hygiene or 90% gloving compliance for any moment except moment 1 in the standard arm. Within the GloveCare arm, hand hygiene compliance was higher compared to glove compliance in Moment 1. In comparison, glove compliance was higher for Moments 2, 3, and 4 than hand hygiene compliance in the GloveCare arm. This indicates the need for modifications to improve compliance with both hand hygiene and gloving in both arms prior to a multicenter study.
- What are the implications of the feasibility findings for the design of the main study?
  We have validated a methodology for glove compliance metrics for a future study. We were also able to establish the event rate of 10%, which will be used to inform the sample size calculation for a future trial, and consider narrowing to the most appropriate patient population with the NICU for this intervention.

## Introduction

Late-onset infection (LOI, defined as infection after 72 h of age) is a serious cause of long-term morbidity and mortality among patients in the neonatal intensive care unit (NICU). As healthcare worker hands are the most common vehicle for transmission of pathogenic organisms to neonates [1], hand hygiene is reported as the most important infection control practice for preventing cross-transmission of microorganisms [2–4]. However, hand hygiene compliance rates in the literature vary immensely (from 5 to 89% [5]) and hand hygiene strategies alone have not shown an association with reduced bloodstream infection rates [6, 7]. Clinically relevant bacteria remain on health care providers’ hands despite hand hygiene [8]. This suggests another barrier, such as non-sterile gloves, may be important to prevent infection.

A proactive approach to prevent, rather than treat LOI, with the risk of neurodevelopmental impairment and antimicrobial resistance, decreases the length of hospital stay and costs of care [9–11]. Adherence to infection control practices in the NICU is fundamental to reduce late-onset infection [12]. Implementation of infection control bundles over 15 years has significantly reduced NICU LOI rates [13]. The primary components of these infection control bundles include collaborative team-based education, central line insertion bundles, and hand hygiene promotion; these are already standard of care in most NICUs [13–17].

The addition of non-sterile glove-based care to standard infection control practices including hand hygiene has been explored. One randomized controlled trial (RCT) demonstrated a significant reduction in gram-positive bloodstream infections in neonates randomized to the non-sterile glove use arm [18]. Additionally, two retrospective studies demonstrated a significant reduction of LOI with non-sterile glove use. Ng et al. demonstrated a nearly 3-fold reduction in late-onset bloodstream infections in preterm infants with gloving compared to conventional hand hygiene alone [6]; however, other practice changes in infection control may have been a confounding factor. Another retrospective cohort in late preterm infants (32–36 weeks gestational age) found 6 episodes of late-onset clinical infection in 111 patients in the standard arm (2.99/1000 hospital days), compared to zero episodes in 89 patients in the gloving arm, with no difference in culture-positive bloodstream infections (2 vs. 0) between groups [19]; however, this study was limited by its small sample size and use of “clinically diagnosed” sepsis. Kaufman et al. had previously published an individually randomized control trial (RCT) which demonstrate a significant reduction in gram-positive bloodstream infections, and fewer central line infections indicating the importance of skin flora as pathogens for neonates.

A key knowledge gap remains in understanding the effectiveness of gloving in preventing both sterile site (e.g., bloodstream) and non-sterile site (e.g., pneumonia) LOI, across various gestational ages and birth weights. In addition, in many of the referenced studies, gloving compliance was rarely reported and/or compared to hand hygiene compliance. Thus, we sought to first determine the feasibility of conducting a multicentre RCT to determine whether glove-based care (in addition to hand hygiene) would reduce LOI in the NICU, as compared to standard care. The objective of our pilot RCT was to assess feasibility outcomes including participant enrolment, hand hygiene compliance, compliance to gloving, and LOI prevalence and rate, to assess if a pragmatic multicentre cluster RCT could be conducted. Additional outcomes include the types of LOI pathogens detected, additional prevalence of patients who had additional precautions (isolation), the LOI adjudication process, and qualitative description of the auditing verification process.

## Materials/patients and methods

This pilot was a single-center study comprising two groups of infants, each recruited sequentially over two separate time periods to receive standard hand hygiene care (Arm 1) or glove-based care (Arm 2) in preparation for a multi-center cluster-crossover trial which will randomize entire sites (clusters) to the two arms and then crossover to the other arm after a period of time. The pilot was conducted in the NICU at McMaster Children’s Hospital (MCH) in Hamilton, Canada, between June 2017 and June 2018. The protocol was registered at www.clinicaltrials.gov (NCT03078335).

### Ethics

The study was approved by the Hamilton Integrated Research Ethics Board (#2175). A waiver of consent process was used as the intervention was considered a minimal risk (per The Interagency Advisory Panel on Research Ethics 2) and the study would not have been feasible without unit-wide standardization of infection prevention and control (IPAC) practices. Bedside nursing staff informed and provided an information sheet to parents about the change in IPAC policy in the NICU for the study duration. Parents had the option to withdraw from data collection within the study, but were cared for based on the arm of randomization during the neonates’ admission. Due to the minimal risk of the intervention itself, no trial-stopping rules or interim analyses were planned.

### Study population and setting/inclusion and exclusion criteria

Infants admitted to the MCH Level 3 NICU for a minimum of 2 days were included in the study for their duration of stay. Any infection outcomes that occurred in infants requiring droplet and/or additional precautions for infection control reasons were excluded from analysis (as this involved glove use for reasons unrelated to the study intervention) as established a priori. Infection outcomes in patients transferred in and out of the NICU from other centers were included for their duration within our facility (events occurring within the first 48h after transfer to our NICU were excluded as they could be attributed to the transferring facility). Any events within 48 h after discharge were not collected as we did not have access to data from external facilities. The MCH NICU hand hygiene compliance prior to the study exceeded the 90% target for moments 1 and 4. Gloves of different sizes and alcohol-based hand rub were available at each bedside and hand hygiene sinks were located in proximity to all bedsides. Per unit standards, upon entry to the NICU, all health care providers were required to scrub to the elbows and there was a “bare below the elbows” policy in the NICU.

### Study design

This single-center randomized pilot study was conducted to evaluate the feasibility of a future multicenter cluster-crossover randomized trial. This pilot study had 2 intervention periods lasting 6 months each, with a 2-week washout period in between. The 2-week washout period ensured provider education was adequately delivered and minimized contamination of neonates that were cared for in both arms of the study. The NICU was randomized using a computer simple randomization sequence (operationalized by an independent statistician) resulting in the standard arm being selected for the first 6-month period, followed by the GloveCare arm. Due to the pragmatic nature of the trial, other clinical practice changes that occurred during the study year were monitored but not deferred. The use of probiotics was introduced as an accepted standard of care in the NICU at the end of the standard arm for necrotizing enterocolitis prevention (Appendix 1). No other major known confounders or infection control/clinical practice changes occurred during the study. The study was open label as health care workers could not be blinded to the treatment assignment.

### Definition of late-onset infection

Late-onset infection episodes were categorized: sterile-site LOI (i.e., culture-positive meningitis, bacteremia, urinary tract infection) and non-sterile-site LOI (culture-negative meningitis, single blood culture positive with coagulase-negative staphylococci, abdominal infection, pneumonia, clinically diagnosed cellulitis, and “culture-negative” sepsis). See Appendix 2 for further details of infection definitions. We also collected C-reactive protein (CRP) measurements and time to the first infection.

### Prior to intervention (standard arm)

Healthcare workers provided standard care, namely hand hygiene before all patient, bed space, and intravenous catheter contact. Information sessions were offered to all clinical staff to review hand hygiene best practices, appropriate glove selection, donning and doffing procedures, and skin care. These sessions occurred prior to study commencement to ensure front-line clinician buy-in to the process/study. During the standard arm, gloves remained accessible to staff as per standard of care, and staff continued to use gloves as they deemed necessary (e.g., prior to contact with body fluids).

### Study intervention period (GloveCare arm)

Refresher educational information sessions (as above) were held again prior to commencing the GloveCare arm (Appendix 3). During the study period, all healthcare workers in the NICU were instructed to wear non-sterile gloves, after routine hand hygiene, for all patient and line contact (GloveCare). Health care providers included any hospital staff providing hands-on care. Multi-disciplinary consultation occurred prior to study commencement to ensure staff were aware of the study, to stress the importance of hand hygiene even during the GloveCare period, and increased auditing measures. During these sessions, strategies to optimize ability to glove during care were discussed. It was also emphasized *a priori* that patient care needs took precedence over the need to don gloves in emergent situations. Patient contact was defined as any contact with the patient or the patient environment (i.e., cot/incubator and any equipment attached to the patient). Intravenous catheter contact was defined as contact with central or peripheral catheters, including making or breaking a connection with the line. Signage and study logos were posted to remind care providers of the GloveCare intervention, as well as the study logo visible at the entrances to the incubators or cots (Appendix 4). Staff were asked to self-report any missed gloving opportunities (and the reasons for) on a data collection sheet available at each bedside (Appendix 4). Parents and caregivers were not included in the GloveCare intervention. While it is recognized that parents may contribute to the colonization of infants with bacteria that may lead to healthcare-associated infections, they were not required to wear gloves because (1) skin to skin contact is considered a critical part of bonding for infants and parents [20], (2) parents provide less hands-on contact with invasive devices, (3) parents are less likely colonized with pathogenic flora [21], and (4) normalization of household bacterial flora is recognized as an important part of the diversification and maturation of the neonatal microbiome [22, 23].

Feasibility outcomes included:

- *Participant enrollment* (target >90% enrollment (e.g., do not sign the opt out of data collection waiver))
- *Hand hygiene compliance* for moments 1 to 4 (target >90% compliance based on weekly randomized audits for moment 1 and moment 4) (moments are defined below)
- *Glove use compliance* during the GloveCare arm (with a target of >90%). The hand hygiene target is higher than the overall hospital-wide hand hygiene target of 80%.

#### Hand hygiene and glove compliance

Compliance with hand hygiene was monitored by auditors three times weekly as per a randomized schedule in both study arms. Hand hygiene is an integral part of care, regardless of the use of gloves. Audits were for a minimum of 1 h, either during day or night shifts (day shift: 7am to 7pm, night shift: 7pm to 7am) to get a representative sample of clinical care and staff (Appendix 6). Randomization of monitoring was performed using SAS Software Version 9.3 (Cary, NC, USA). Moments 1 to 4 as defined by Public Health Ontario [24] were audited using licensed Handy Audit® (Version 2.0) software. The results of the hand hygiene and glove compliance audits were relayed back to the NICU on a monthly basis during routine IPAC meetings with the NICU staff to continue to support adherence with study interventions. We also allowed providers to self-report in a separate document when they had a known glove miss during the gloving arm, to understand in what circumstances were providers finding gloving difficult to comply with.

For glove compliance, we defined Moment 1 as donning gloves before touching the patient or their environment, Moment 2 as donning gloves before an aseptic procedure, Moment 3 as doffing gloves after contact with body fluid, and Moment 4 as doffing gloves after touching patient or patient environment. We developed a Python script to measure the four moments of glove compliance from Handy Audit® reports. An infectious disease clinician and hospital hand hygiene coordinator manually assessed the four glove compliance moments for 5% of all audits and compared them to the results of the Python script for validation agreement. From the Python script glove compliance results, we manually removed Moment 3 and 4 glove misses when there was no donning prior; thus, only a Moment 1 miss would be recorded. Overall glove compliance was then calculated from all audits during the glove compliance period. Glove compliance was measured in addition to hand hygiene compliance; staff must perform hand hygiene before donning gloves and after doffing gloves as this is the standard of care.

The primary clinical outcome was *the event rate of LOI*. LOI adjudication was performed to ensure accurate event rates by two independent adjudicators blinded to study arm (SK, KT). The adjudication process included review of the clinical chart including clinical notes, laboratory, and microbiology data. The 6-month duration of each arm in the study was selected as the planned duration for the multicenter trial depending on the event rate of infection episodes during the pilot. Study process outcomes include the adequacy of research resource allocation, research coordinator capacity, processing times for evaluating a potential new event of LOS, time required for adjudication of events, and ensuring the accuracy of hand hygiene compliance data.

### Data collection

Data was recorded in an anonymized Research Electronic Data Capture Program (REDCap ®) database [25]. Information on each neonate including demographics, infection risk factors (i.e., duration of rupture of membranes, prenatal steroid use, mode of delivery, vascular access, ventilatory support, immune-suppressing medications, nutrition), admitting facility, length of stay, and duration of isolation precautions. Infection episodes were collected retrospectively by chart review of all cases that received antibiotics for more than 3 days. Each event was adjudicated by two co-authors blinded to study arm (SK, KT) using a priori determined criteria for infection episodes.

### Sample size

As this was a feasibility study, we took a convenience sample of our single site as the sample size. We did not power this study to see a difference in infection rate; however, we did plan for exploratory analysis to assess changes in infection rate based on study arm, recognizing we are not powered to see a difference.

### Statistical analysis

Feasibility, clinical, and process outcomes were tabulated and descriptively compared between neonates in the GloveCare and standard arm. Hand hygiene compliance between GloveCare and standard arm was compared using estimated odds ratios accompanied by 95% confidence intervals (CI), and *p*-value calculated through the chi-square test, for all four moments of hand hygiene. Likewise, we calculated gloving compliance. Other characteristics, including the demographic description of our study participants, the distribution of pathogens, event rate of LOI, and prevalence and duration of additional precautions, were descriptively compared between both arms (Tables 1, 2, and 3). Categorical data is presented as frequency (*n*) and proportion (%) and analyzed using chi-squared tests or Fisher’s exact test as appropriate. Continuous data which is normally distributed is presented as mean and standard deviation (SD) as appropriate and analyzed using independent *t*-tests. *P*-values less than 0.05 are considered statistically significant.

**Table 1:** Pathogen distribution of late-onset infection events

Number of primary late-onset infection events and mortality	GloveCare arm *N*=63	Standard arm *N*=48	***P***-value (chi-square test)
**Sterile site infections,***n* (%)			
Any sterile site infection (culture positive meningitis, bacteremia, urinary tract infection)	14 (22.2)	15 (31.3)	0.284
Gram positive	9	7	0.965
Gram negative	9	5	0.264
Meningitis (*n* (%))	3 (4.8)	0 (0.0)	0.257 (FE)
Gram-positive	2 (66.7)		
Gram-negative	1 (33.3)		
Mean duration of antibiotic therapy (SD)	29.33 (16.4)		
Mean C-reactive protein (CRP, mg/L) (SD)	124.25 (72.1)		
Bacteremia (*n* (%))	4 (6.3)	6 (12.5)	0.324 (FE)
Gram-positive	3 (75.0)	0 (0.0)	0.257 (FE)
Gram-negative	1 (25.0)	5 (83.3)	0.0831 (FE)
Candida	0 (0.0)	1 (16.7)	
Mean duration of antibiotic therapy (SD)	14.25 (9.4)	30.50 (26.5)	
Mean C-reactive protein (CRP, mg/L) (SD)	81.05 (81.7)	139.42 (177.0)	
Urinary Tract Infection (*n* (%))	7 (11.1)	9 (18.8)	0.256 (CS)
Gram-positive	4 (57.1)	7 (77.8)	0.203 (FE)
Gram-negative	3 (42.9)	2 (28.6)	1.00 (FE)
Mean duration of antibiotic therapy (SD)	9.29 (3.6)	14.44 (12.3)	
Mean C-reactive protein (CRP, mg/L) (SD)	17.36 (15.7)	21.70 (14.3)	
**Nonsterile site infections,***n* (%)			
Secondary infection outcomes	49 (77.8)	33 (68.8)	0.284
Culture negative meningitis	3 (4.8)	0 (0.0)	0.257
Mean duration of antibiotic therapy (SD)	26.67 (16.3)		
Mean C-reactive protein (CRP, mg/L) (SD)	30.67 (39.2)		
Single positive CONS blood culture (*n* (%))	11 (17.5)	4 (8.3)	0.164
Mean duration of antibiotic therapy (SD)	9.27 (3.3)	10.50 (4.8)	
Mean C-reactive protein (CRP, mg/L) (SD)	41.42(47.6)	15.07 (24.1)	
Abdominal infection (*n* (%))	4 (6.3)	7 (14.6)	0.203
Mean duration of antibiotic therapy (SD)	14.75 (9.0)	9.86 (5.2)	
Mean C-reactive protein (CRP, mg/L) (SD)	114.00 (91.5)	68.91 (93.9)	
Pneumonia (*n* (%))	9 (14.3)	6 (12.5)	0.785
Mean duration of antibiotic therapy (SD)	9.89 (1.8)	10.67 (6.1)	
Mean C-reactive protein (CRP, mg/L) (SD)	34.28 (23.0)	47.15 (55.1)	
Cellulitis (*n* (%))	2 (3.2)	0 (0.0)	0.505
Mean duration of antibiotic therapy (SD)	9 (1.4)		
Mean C-reactive protein (CRP, mg/L) (SD)	51.60 (17.7)		
Culture negative sepsis (*n* (%))	20 (31.7)	16 (33.3)	0.860
Mean duration of antibiotic therapy (SD)	8.30 (3.4)	8.75 (2.8)	
Mean C-reactive protein (CRP, mg/L) (SD)	52.04 (43.3)	47.87 (76.6)	
Time to first infection, among all patients with infection, days (*n*=75), mean (sd) (“Removing” time in additional precautions)	20.1 (16.1)	11.4 (8.3)	**0.004**

*FE* Fisher’s exact test used instead of chi-square

**Table 2:** Event rate of different infection events

Clinical measure	GloveCare arm (***N***=360)	Standard arm (***N***=390)	Overall (***N***=750)
Total 1^st^ episode infections (*n*, %)	41 (11.4)	34 (8.7)	75 (10.0)
Sterile site 1^st^ episode infections (*n*, %)	11 (3.1)	10 (2.6)	21 (2.8)
Nonsterile site 1^st^ episode infections (*n*, %)	30 (8.3)	24 (6.2)	54 (7.2)
Person-days	7686	7162	14848
Number of total episodes	63	48	111
Total episodes/1000 person-days)	8.20	6.70	7.46
Number of sterile-site episodes	14	15	
Sterile-site episodes/1000 person-days	1.82	2.09	
Number of nonsterile-site episodes	49	33	
Nonsterile-site episodes/1000 person-days	6.38	4.61

**Table 3:** Infants entered into additional isolation precautions

Additional precautions	GloveCare arm (***N***= 360)	Standard arm (***N***=390)	***P***-value (chi-square)
Once, *n* (%)	26 (7.2)	36 (9.2)	0.318 (CS)
Twice, *n* (%)	0 (0.0)	7 (1.8)	0.0157 (FE)
Thrice, *n* (%)	0 (0.0)	3 (0.8)	0.140 (FE)
Four times, *n* (%)	0 (0.0)	2 (0.5)	0.270 (FE)
Episode 1, mean (sd), median	18.00 (25.7), 6.50	7.75 (15.5), 4.00	0.249
Episode 2, mean (sd), median	**--**	0.69 (2.7), 0.00	
Episode 3, mean (sd), median	**--**	--	
Episode 4, mean (sd), median	**--**	--	
Total duration, mean (sd), median	18.0 (25.7), 6.5	8.4 (15.6), 4.0	0.100

*CS* chi-square, *FE* Fisher’s exact

## Results

### Demographic characteristics and recruitment

**Fig. 1:**
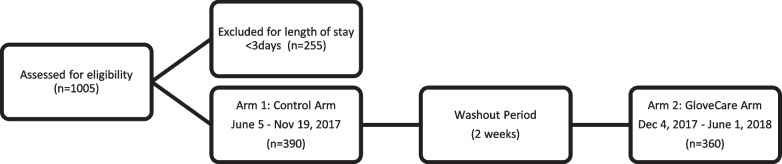
Flowchart of enrollment, randomization, and analysis

During the enrollment period (June 5, 2017, to June 1, 2018), 1005 neonates were assessed for eligibility, and 255 were excluded due to short length of stay (less than 3 days) (Fig. 1). We enrolled 390 neonates in the standard arm (June 5, 2017, to November 19, 2017) and 360 neonates in the GloveCare arm (December 4, 2017, to June 1, 2018). Demographic information on the two groups is included in Table 4. There was no statistically significant difference in the prevalence and duration of additional precautions (Table 3). No patients opted out of data collection (Table 5). Probiotics (Florababy ™) was introduced for necrotizing enterocolitis prevention at the end of the standard arm (Nov 28, 2017).

**Table 4:** Study demographic and clinical characteristics

Demographic and clinical characteristics	GloveCare arm (***N***=360)	Standard arm (***N***=390)	***P***-value
Male gender, *n* (%)	208 (57.8)	238 (61.0)	0.365
Gestation at birth (weeks), mean (sd)	34.11 (4.7)	34.41 (4.8)	0.396
Birth weight (grams), mean (sd)	2332.03 (35.7)	2452.76 (95.5)	0.122
Apgar score (1 min), mean (sd)	6.53 (2.5)	6.69 (2.4)	0.375
Apgar score (5 min), mean (sd)	7.88 (1.7)	7.94 (1.6)	0.624
**Admitted from**			0.369 (FE)
Labour and delivery, *n* (%)	257 (71.4)	281 (72.1)	
Postpartum ward, *n* (%)	6 (1.7)	11 (2.8)	
Community level 2 nursery, *n* (%)	93 (25.8)	97 (24.9)	
Home birth, *n* (%)	4 (1.1)	1 (0.3)	
Vaginal delivery, *n* (%)	168 (46.7)	177 (45.4)	0.725
**Rupture of membranes (ROM)**			
Ruptured, duration known, *n* (%)	351 (97.5)	388 (99.5)	**0.024**
Duration of ROM, mean (sd), among ROM known (glove: *n*=351; standard: *n*=388)	28.93 (132.0)	36.47 (172.4)	0.508
**Prenatal steroid use (in <34 week),***n* (%) (*N*=340)	118 (71.5)	131 (74.9)	
**Risk factors for infection prior to first episode of LOI**			
Central venous line (CVL), *n* (%)	156 (43.3)	168 (43.1)	
Peripheral IV access, *n* (%)	277 (76.9)	300 (76.9)	
Mechanical ventilation, *n* (%)	108 (30.0)	102 (26.2)	
Continuous positive airway pressure (CPAP), *n* (%)	212 (58.9)	263 (67.4)	0.283
Neonatal steroid (hydrocortisone), *n* (%)	19 (5.3)	26 (6.7)	**0.044**
Acid inhibition, *n* (%)	17 (4.7)	27 (6.9)	0.086
Ventricular shunt, *n* (%)	0 (0.0)	2 (0.5)	0.373
Surgical PDA ligation, *n* (%)	0 (0.0)	1 (0.3)	0.487
Total parenteral nutrition (TPN), n (%)	261 (72.5)	282 (72.3)	0.944
Duration of TPN, mean (sd)	7.76 (13.7)	7.42 (11.4)	0.995
Human milk, *n* (%)			0.241
Only	143 (39.7)	180 (46.2)	
Any	173 (48.1)	173 (44.4)	
None	44 (12.2)	37 (9.5)	
Probiotics, *n* (%)	156 (43.3)	9 (2.3)	**0.015**
Length of stay in the NICU, days, mean (sd) (Includes isolation time)	22.7 (29.1)	19.1 (25.2)	0.079
Total duration of isolation, patient-days	468	304	

*FE* Fisher’s exact test, used where chi-square tests not doable due to small sample size, *ROM* rupture of membrane, *IV* intravenous, *CVL* central vascular access, *CPAP* continuous positive airway pressure, *PDA* patent ductus arteriosus, *TPN* total parenteral nutrition, *NICU* neonatal intensive care unit

**Table 5:** Primary feasibility outcome

Outcome	Target	Results
Participant enrolment (*N* (%))	>90%	1005 (100%)
Event rate of LOI—number of patients (*N* (%))	Not applicable	75 (10%)
Adequacy of resource allocation	1 FTE Research coordinator	Required extra data entry support
Processing time for evaluating new LOS	Not applicable	Estimated at 5 to 30 min depending on complexity of LOI
Adjudication of LOS (mean (range))	Not applicable	20 minutes per case (3–45 min)
Accuracy of data collection	Not applicable	Adjudication resulted in exclusion of 124 LOIs
Hand hygiene compliance moment 1	>90% in both arms	87.03% in standard arm, 78.28% in GloveCare arm
Hand hygiene compliance moment 4	>90% in both arms	87.37% in standard arm 80.76% in GloveCare arm
Glove compliance moment 1	>90% in GloveCare arm	66.2% in GloveCare arm
Glove compliance moment 4	>90% in GloveCare arm	83.30% in GloveCare arm

*LOI* late onset infection, *LOS* length of stay

### Hand hygiene and gloving compliance

Hand hygiene compliance in the GloveCare Arm was lower than the standard arm across all moments of hand hygiene, with statistically significant differences seen in Moment 1, Moment 4 overall, and Moment 4 touch patients (Table 6). However, the results should be interpreted with caution; we cannot know which healthcare workers were evaluated at each observation and thus we cannot consider each hand hygiene moment as an independent observation. We did not achieve our target of 90% hand hygiene or 90% gloving compliance for any moment except moment 1 in the standard arm. Within the GloveCare arm, hand hygiene compliance was higher compared to glove compliance in Moment 1. In comparison, glove compliance was higher for Moment 2, 3, and 4 than hand hygiene compliance in the GloveCare arm.

**Table 6:** Hand hygiene and gloving compliance outcomes

	Hand Hygiene compliance in standard arm—number of HH audits *n* (%)	Hand hygiene compliance in GloveCare arm—number of HH audits *n* (%)	Hand hygiene compliance: standard vs GloveCare Odds ratio (95% CI)	*P*-value	Glove compliance in GloveCare arm *n* (%), *p*-value	Compliance in GloveCare arm: hand hygiene vs gloving Odds ratio (95% CI), *p*-value	*P*-value
**Moment 1 Total**	510 (87.03)	346 (78.28)	1.86 (1.34, 2.59)	<0.001	525 (66.20)	1.83 (1.40, 2.41)	<0.001
Moment 1 touch patient	266 (90.80)	194 (80.83)	2.33 (1.40, 3.89)	<0.001	371 (78.60)	1.14 (0.77, 1.69)	0.487
Moment 1 Touch patient environment	244 (83.28)	152 (75.25)	1.63 (1.05, 2.55)	0.028	154 (47.98)	3.30 (2.24, 4.85)	<0.001
**Moment 2**	56 (40.58)	28 (28.28)	1.73 (1.00, 3.01)	0.051	75 (52.45)	0.36 (0.21, 0.62)	<0.001
**Moment 3**	97 (78.86)	111 (77.08)	1.11 (0.62, 1.98)	0.727	108 (85.71)	0.56 (0.30, 1.06)	0.071
**Moment 4 Total**^**a**^	844 (87.37)	634 (80.76)	1.65 (1.27, 2.14)	<0.001	409 (83.30)	0.84 (0.63, 1.13)	0.254
Moment 4 touch patient	353 (86.31)	434 (79.49)	1.63 (1.15, 2.31)	0.006	285 (84.57)	0.71 (0.49, 1.01)	0.059
Moment 4 touch patient environment	286 (84.37)	312 (84.55)	0.99 (0.66, 1.48)	0.945	124 (80.52)	1.06 (0.61, 1.84)	0.258

^a^Hand hygiene compliance in GloveCare arm Moment 4 does not add up because touch patient and touch patient environment can be duplicate events

Recruitment was 100% with no families choosing to opt out of data collection. LOI event rate for this study was 10%.

### Barriers to gloving compliance

During the GloveCare arm, staff self-reported inability to comply with gloving on 49 occasions, the most common reason documented was having forgotten (96%) (Appendix 5, Table 7). Reasons for missed glove opportunities described by staff in informal interviews were that gloves required another step (e.g., de-gloving, hand hygiene, re-gloving); this may have been an additional barrier to hand hygiene in the GloveCare arm. In some scenarios, staff described that gloving was not possible due to urgent care needs and being unable to delay care to don gloves.

### Validation of glove compliance data

While Handy Audit® was able to collect when an individual donned or doffed gloves, it was unable to interpret this information into overall glove compliance metrics. Auditing and validating glove compliance data became a feasibility outcome post hoc. We developed a Python® script to convert an individual’s don and doffing activity into four Moments of glove compliance, similar to hand hygiene. A hand hygiene coordinator and infectious disease clinician iteratively validated the Python script for over 200 h. Every iteration included manually assessing 5% of all audits and comparing them to glove compliance report from the Python script. Validation of each audit and glove compliance report required a range of 3 to 20 min depending on the complexity of the care provided. Validation was considered complete when there were no discrepancies between the manual auditing and Python script results. Our self-report documentation on glove misses was rarely used and did not provide added value to our compliance data.

### LOI adjudication process

LOI adjudication was performed to ensure accurate event rates. The process required on average 20 min per case, with a range of 3 to 45 min. The LOI adjudication process excluded 124 LOIs: 6 because they occurred while patients were in additional precautions, 24 because they occurred within the first 3 days of birth (and therefore were early-onset infection), and 94 because they did not meet criteria upon adjudication (e.g., antibiotics discontinued within 48 h).

### LOI prevalence and rate

The overall prevalence of first-episode LOI in the entire study period was 10%. There was a LOI prevalence of 11.4% in the GloveCare arm and a prevalence of 8.7% in the standard arm %. The event rate was also higher in the GloveCare group, which showed 8.2 LOI episodes per 1000 person-days compared to the standard group of 6.7 LOI per 1000 person-days [incidence rate ratio = 1.22, 95% CI = (0.84, 1.78), *p*-value=0.293]

## Discussion

This pilot study evaluated the feasibility of the GloveCare in terms of hand hygiene compliance, gloving compliance, adjudication of LOI outcomes, event rate, and resources required to validate compliance data. Overall, this study affirms that GloveCare is a feasible intervention for a multicenter cluster RCT. The results indicated that we must address both hand hygiene and gloving fidelity, and ensure compliance data are collected accurately and efficiently, at the outset of a future multicentre RCT.

We demonstrated recruitment feasibility by exceeding our targeted enrollment rate of 90% and achieved 100% with no families choosing to opt out of data collection. We were able to ascertain the LOI event rate for this NICU at 10% which will greatly inform the sample size calculation for a future multicenter RCT. We will also use the variance observed from the pilot to inform the predictions about the variance in the outcome for the larger trial. This incidence is within the range expected in the literature [6, 18].

The hand hygiene and glove compliance targets of 90% were not achieved for either treatment arm of this study across all four moments and may have been unrealistic targets, based on previous studies by Kaufman et al. (79% hand hygiene compliance) [18] and Baloh et al. (42% hand hygiene compliance) [26]. Hand hygiene compliance was also markedly lower in the GloveCare arm. This could possibly be due to the belief that hand hygiene is not necessary if donning gloves, despite educating staff about hand hygiene and gloving best practices. Furthermore, if providers perceived each other to be less compliant with hand hygiene while gloving, then they may be more likely to investigate nonspecific symptoms in infants as possible sepsis, thereby leading to a higher event rate of more subjective events (e.g., culture-negative sepsis) during the GloveCare arm. Finally, we identified barriers to gloving through the self-reported tool and staff feedback. This will inform the orientation plan of future studies.

An additional methodologic limitation included the inability to assess if the difference in hand hygiene compliance between treatment arms was statistically significant, due to the absence of data on individual healthcare workers. Consequently, we could not adjust for inadequate hand hygiene as a confounder. A limitation of hand hygiene auditing processes used in most studies is the potential for Hawthorne effect [27, 28], which should falsely elevate compliance rates above true compliance due to visible observers. Other hand hygiene auditing methods include peer auditors, video technology, or validated counts of alcohol-based hand rub uses based on anticipated care environments [29–32]. However, these techniques were not feasible to implement in our setting, due to the resource intensity and patient and provider privacy concerns. We felt that using independent auditing, the gold standard for hand hygiene monitoring, was the optimal approach. We will likely not use any self-report documentation of known misses in gloving given it had poor uptake overall in the pilot.

Secondly, future studies may consider limiting themselves to assessing patients who are low birthweight (i.e., <1500 grams) to gain a higher event rate, and thus, require less sample size for achieving sufficient statistical power. A further challenge for future studies is that while sterile site and nonsterile site infections differ markedly in patient importance, examining them separately would greatly increase the required sample size, as sterile site infections are rare.

Thirdly, validating glove compliance metrics was a time-intensive challenge. Numerous glove compliance scenarios required discussion and consensus between an infectious disease physician and a hand hygiene coordinator. To ease this process, perhaps all possible gloving scenarios and rules should be established prior to script development; this could be incorporated into a future study. A limitation of our validation process is that it relies on Handy Audit® results and must be further tested using outputs from other hand hygiene compliance software.

In planning for the future multi-center trial, we will likely pursue a cluster crossover trial so each center acts as its own control. Our event rate of around 10% was on the lower range of reported late-onset infections which can range between 10 and 30% depending on the gestation ages of the infants. Sample size calculations would likely be based around the lower range given the pragmatic nature of enrolling the entire NICU rather than just the preterm infants would be required. Prior to enrolling centers, we would want to review their relative late-onset infection rates as well to be able to plan for the number of centers required. Our study team felt that a 15% reduction in the incident rate ratio late-onset infection would be clinically significant, and a secondary outcome of delay to time to infection would be another outcome of interest recognizing the highest risk period for NICU infants is their first weeks of life. It will be important to ensure education around hand hygiene for both arms of the study is optimized, and within a multi-center study subgroup analysis among sites with higher and lower hand hygiene compliance may improve our understanding of the impact this has on evaluating the effectiveness of glove based care to prevent LOI.

## Conclusion

Our pilot study has demonstrated the feasibility of GloveCare. We have also shown that the feasibility of conducting a multicenter cluster randomized control trial in the future to assess the efficacy of non-sterile gloves to prevent LOI in the NICU is feasible. Additionally, this pilot study demonstrated that a waiver of consent for such studies is a feasible recruitment approach for similar low-risk interventions. Our feasibility target for compliance was not met during this study; however, the pilot study greatly informed the validation required for auditing glove compliance for a future study. Improving hand hygiene compliance efforts in the GloveCare arm will be critical to understand the potential impact of gloves. Lower hand hygiene rates in the GloveCare arm may have inhibited the effectiveness of gloving in reducing infections. Overall, the current pilot study has informed researchers in developing and executing a higher quality future multi-center trial.

## Data Availability

The datasets during and/or analyzed during the current study are available from the corresponding author on reasonable request.

## Appendix 1

### Adjudication criteria and procedure for late onset infection events

Potential events of late-onset infection were flagged in patients admitted to the NICU for more than 2 days with microbiologic clinical cultures collected. Events were adjudicated based on retrospective review of clinical charts, microbiology, and infection control surveillance hospital epidemiologists.

Late-onset infection events met the following criteria:

1. Occurring after 2 days of life to exclude early-onset infection events related to in utero environments or delivery
2. Had at least 2 compatible signs and symptoms (including temperature instability, hemodynamic changes, respiratory distress, and increased inflammatory markers (CRP and WBC based on gestation age cut offs [33], change in feeding tolerance, or lethargy as documented by the treating team)
3. Had at a minimum; blood cultures sent for analysis
4. Antibiotic treatment for more than 4 days to eliminate inclusion of episodes of suspected but not clinically or microbiologically proven infection as no clinically significant infections are treated for <4 days

Late-onset infection episodes were prioritized based on severity, in the following order:

A) Primary outcomes (Sterile-site LOI)
  1. Culture-positive meningitis
  2. Bacteremia (positive blood culture, two positive blood cultures for coagulase-negative staphylococci)
  3. Urinary tract infection (positive urine culture, clinically diagnosed UTI with positive urinalysis (nitrites, leukocytes))
B) Secondary outcomes (non-sterile-site LOI)
  1. Culture-negative meningitis (CSF pleocytosis (WBC>20, and cultures negative as often empirically started on antibiotics prior to CSF collection)
  2. Single blood culture positive with coagulase-negative staphylococci
  3. Abdominal infection (includes necrotizing enterocolitis, spontaneous intestinal perforation, and intra-abdominal collection)
  4. Pneumonia (clinically determined based on CXR findings and endotracheal tube cultures)
  5. Clinically diagnosed cellulitis
  6. Culture-negative sepsis (negative blood cultures with consistent symptomatology as defined above)

## Appendix 5

Table 7

**Table 7:** Self-reporting of episodes of non-compliance with gloving was also implemented with a bedside charting tool

Date	# of times this shift	Reason (check/comment				
		Acuity	Tape	Forgot	palpate	Other
**11/20/17**	3	√	√	√		

## Appendix 6

Table 8

**Table 8:** Randomization table of study arms and hand hygiene audits

Weekly shifts: Random Seed: 6321							
Period	Treatment group						
1	Usual care						
2	Gloves + usual care						
Weekly shifts: Random Seed: 6321							
**Week**	**Date (2017–2018)**	**Shift 1**	**Auditor 1**	**Shift 2**	**Auditor 1**	**Shift 3**	**Auditor 1**
1	June 5–June 9	Mon AM		Mon PM		Wed PM	
2	June 12–June 16	Wed AM		Fri PM		Thurs AM	
3	June 19–June 23	Mon AM		Thurs PM		Thurs AM	
4	June 26–June 30	Tues AM		Fri AM		Fri PM	
5	July 3–July 7	Thurs AM		Thurs PM		Wed PM	
6	July 10–July 14	Mon PM		Mon AM		Fri PM	
7	July 17–July 21	Thurs PM		Thurs AM		Wed PM	
8	July 24–July 28	Wed PM		Mon PM		Thurs AM	
9	July 31–Aug 4	Wed AM		Fri PM		Mon PM	
10	Aug 7–Aug 11	Fri PM		Mon AM		Tues AM	
11	Aug 14–Aug 18	Wed PM		Fri PM		Tues PM	
12	Aug 21–Aug 25	Fri AM		Tues AM		Mon AM	
13	Aug 28–Sept 1	Fri PM		Thurs AM		Wed PM	
14	Sept 4–Sept 8	Tues PM		Tues AM		Thurs AM	
15	Sept 11–Sept 15	Tues PM		Wed PM		Thurs PM	
16	Sept 18–Sept 22	Wed AM		Thurs PM		Wed PM	
17	Sept 25–Sept 29	Thurs PM		Wed PM		Mon AM	
18	Oct 2–Oct 6	Wed AM		Fri AM		Fri PM	
19	Oct 9–Oct 13	Tues AM		Mon PM		Fri AM	
20	Oct 16–Oct 20	Mon PM		Fri PM		Tues PM	
21	Oct 23–Oct 27	Tues AM		Wed AM		Tues PM	
22	Oct 30–Nov 3	Tues AM		Thurs AM		Mon AM	
23	Nov 6–Nov 10	Mon PM		Wed PM		Tues AM	
24	Nov 13–Nov 17	Thurs PM		Thurs AM		Mon PM	
25	Nov 20–Nov 24	Wed PM		Mon PM		Thurs PM	
26	Nov 27–Dec 1	Tues PM		Wed PM		Tues AM	
27	Dec 4–Dec 8	Fri PM		Tues AM		Mon AM	
28	Dec 11–Dec 15	Tues PM		Mon PM		Thurs AM	
29	Dec 18–Dec 22	Wed PM		Mon PM		Tues PM	
30	Dec 25–Dec 29	Wed PM		Mon PM		Mon AM	
31	Jan 1–Jan 5	Mon PM		Thurs AM		Thurs PM	
32	Jan 8–Jan 12	Fri PM		Thurs AM		Tues PM	
33	Jan 15–Jan 19	Thurs AM		Fri AM		Tues AM	
34	Jan 22–Jan 26	Thurs PM		Mon AM		Fri AM	
35	Jan 29–Feb 2	Mon AM		Fri AM		Thurs AM	
36	Feb 5–Feb 9	Tues AM		Fri PM		Thurs AM	
37	Feb 12–Feb 16	Tues AM		Mon AM		Wed AM	
38	Feb 19–Feb 23	Thurs AM		Wed AM		Wed PM	
39	Feb 26–March 2	Wed PM		Wed AM		Fri AM	
40	March 5–March 9	Tues PM		Mon AM		Fri AM	
41	March 12–March 16	Thurs AM		Fri AM		Fri PM	
42	March 19–March 23	Tues AM		Fri AM		Wed PM	
43	March 26–March 30	Fri AM		Wed PM		Mon PM	
44	April 2–April 6	Tues PM		Fri AM		Wed AM	
45	April 9–April 13	Tues AM		Mon AM		Wed AM	
46	April 16–April 20	Wed AM		Thurs PM		Fri AM	
47	April 23–April 27	Thurs PM		Tues PM		Mon AM	
48	April 30–May 4	Mon AM		Thurs PM		Thurs AM	
49	May 7–May 11	Thurs PM		Mon AM		Wed AM	
50	May 14–May 18	Fri PM		Tues AM		Thurs PM	
51	May 21–May 25	Wed AM		Thurs AM		Mon PM	
52	May 28–June 1	Tues PM		Fri AM		Thurs AM	
53	June 4–June 8	Fri PM		Thurs AM		Fri AM	
54	June 11–June 15	Fri AM		Thurs AM		Mon AM	
55	June 18–June 15	Wed PM		Thurs PM		Tues AM	
